# Supplementary material for: Quality and Integrated Service Delivery: A Cross-Sectional Study of the Effects of Malaria and Antenatal Service Quality on Malaria Intervention Use in Sub-Saharan Africa
Source: Trop Med Infect Dis. 2022 Nov 9;7(11):363. doi: 10.3390/tropicalmed7110363 (PMC9698472; doi:10.3390/tropicalmed7110363)
Supplement: Supplementary file 1 [file tropicalmed-07-00363-s001.zip › tropicalmed-1954455-supplementary.pdf]

Supplemental Files

Text S1. Quality indicator mapping methods and results

To guide the comprehensive and systematic selection of quality indicators, we constructed a theory-derived quality tool. The tool aligns the quality frameworks of the World Health Organization (WHO) [1] and Donabedian [2], which characterize quality as a multidimensional and multi-domain concept, respectively. We used the tool in conjunction with the previously described methods of Lee et al. 2016 [3] to review the literature, map indicators to the tool, and systematically select parsimonious sets of the quality indicators for MiP services delivered during ANC and ANC quality generally. The tool includes the structure, process, and outcomes domains down the left-hand side and six quality dimensions of effectiveness, efficiency, accessibility, acceptability/patient-centeredness, safety, and equity across the top. Indicators were either categorical or continuous and fell into both a domain and a dimension of quality. We aimed to qualitatively ensure a minimum of 2-3 indicators per dimension, and at least one indicator per domain, where feasible. We constructed Lee's et al. 2016 ANC quality score for each facility as an unweighted average of indicators within each of the five dimensions, excluding equity, and then overall on a continuous scale from 0 to 100. Of the original ANC quality score indicators, we excluded patient satisfaction with an ANC quality that was not available in Tanzania. We followed the same procedure to construct the MiP service quality score. We noted no indicators specifically measuring the dimension equity.

A total of 17 indicators of the MiP service quality and 13 indicators of the ANC quality were selected using the quality tool (Table 1, Table S1) [3]. Although our overall quality scores included a range of structural and process measures to ensure a comprehensive assessment, the results of the initial tool mapping for the MiP quality indicated a relatively more robust emphasis on the capture of process measures compared to the structural in SPA data. By contrast, previously published ANC quality indicators were deliberately structural [3]. Structural barriers, such as stock-outs and user fees, as well as process barriers, such as the failure to routinely deliver IPTp as part of antenatal care, can have a direct effect on the receipt of IPTp and dosing [4], and where possible, we considered the inclusion of measures for these barriers. Additionally, process-related barriers and facilitators for the provision of care as measured through the IPTp uptake included general and specific (e.g., IPTp timing, efficacy, and safety) provider training, correct trimester identification, and easy-to-understand training materials [5-8]. Although many of these factors are not captured in the SPA data, we included two indicators of training on IPTp and pregnancy complications.

Text S2. Multilevel modeling methods and rationale

DHS data were multilevel at the individual, cluster, and region levels where meaning observations within groups were likely to be correlated. Our outcomes were nonrare and dichotomous. As odds ratios are best for approximating risk ratios when the outcome is rare, we employed a suitable alternative, the modified Poisson regression. We calculated crude effects using modified Poisson regression with a robust error variance, which estimates the risks, avoids the overestimation of error by using a sandwich estimator, and is a suitable approach for non-rare outcomes [9]. A modified Poisson regression can also be extended to multilevel data in Stata 14.0. To do so for our adjusted models, we first built three empty models with random effects only to determine whether, for each outcome, a two- or three-level model was most suitable, based on the level of variation present at region and cluster levels. We then built the three adjusted, mixed effects multilevel modified Poisson

models with robust variance estimation and random effects at the cluster and region levels for pooled data to test the relationship between ANC and MiP service quality scores with each outcome of interest.

We reweighted the countries equally in the pooled models using probability weights for the mother or child as appropriate to avoid any single country overly contributing to the results. It was not possible to account for the survey design using the 'svy' suite of Stata commands as DHS data included probability weights at the individual level only. The multilevel modeling of complex survey data requires weights at the cluster level and the re-weighting of lower weight levels in relation to those at higher levels [10]. We instead employed probability weights with standard multilevel modeling with robust variance estimation and adjusted these for residence location in every model irrespective of significance to account for the stratified survey design [11].

We also considered multiplicative interactions as appropriate for multilevel models by generating the interaction term in advance and examining the effect that each component of the interaction term included. To avoid multicollinearity between the interaction terms and continuous variables of interest, we mean-centered continuous variables, including the MiP and ANC quality and used the mean-centered form(s) to generate interaction terms. Once a variable was mean-centered, we used this form in every model to ensure the comparability and consistency of the approach. We used an alpha=0.10 cut-off for inclusion in the adjusted models. For statistically significant interaction terms, we also graphed the adjusted term to visually assess its effect. This was particularly important for continuous by continuous interaction terms using the user-written program 'mfpigen', as the extent of the nonlinear relationships, or lack thereof, might not have been apparent from a coefficient and confidence interval alone [12].

We used pooled findings to evaluate the relationships of interest for the entire sub-Saharan African region where malaria was endemic, under the assumption that the study countries were randomly drawn from and were representative of the region's population. Multilevel, mixed-effects models with dichotomous outcomes required a sufficient sample size not only at the individual level but at higher group levels [13]. We met these requirements using a combination of effect size estimations for the fixed effect Poisson models using reasonable effect sizes for the social science research ranging from 1.5 to 2.0, and a rule of thumb guidance to ensure a sufficient sample size at each model level and for linear regression [14-16].

However, we also conducted and reported priori stratified country analyses using individual probability weights divided by 1,000,000 in-country models and following DHS guidance. These were meant to be hypothesis-generating and thus exploratory in nature. We felt it was important to do so, given the likely heterogeneities in country-specific contexts that may affect the service quality due to a range of factors (e.g., governance structure, funding, and programming). For example, differences in the epidemiological trend for malaria over time could affect a country's performance in terms of the quality of care. Comparing the pooled to a country's findings could also help support the stratified country findings based on smaller sample sizes.

Table S1. **List of variables according to data source.**

	DHS	SPA	MAP	Population
Country	X	X		X
IPTp uptake during pregnancy	X			
ITN use the night prior in pregnancy	X			
ITN use the night prior in children under 5	X			
Child's age in months	X			
Child's gender (female)	X			
Number of ANC visits	X			
Mother's age	X			
Mother's education	X			
Parity	X			
Gestational age	X			
Household wealth quintile	X			
Household size	X			
Survey administered during malaria season	X			
HIV prevalence	X			
Cluster	X			
Region	X	X		X
Malaria endemicity			X	
Residence location (urban/rural)	X			
ANC quality*		X		
MiP quality*		X		
Facility density**		X		X

Abbreviations: DHS: demographic and health surveys; SPA: service provision assessment; MAP: Malaria Atlas Project. Notes: *Indicates indicator is constructed from more than one variable from the same data source; **Indicates indicator is constructed from more than one data source; Population data are regional population estimates available from the national statistics departments for Kenya, Namibia Senegal, and Tanzania.

Table S2. Mapping of antenatal care quality indicators to combined quality framework tool. We mapped Lee's et al. 2016 indicators of antenatal care quality to our adapted quality framework tool. Greyed out indicators were not included in the final quality score due to unavailability in all country data.

		World Health Organization Framework					
		**Effectiveness**	**Efficiency**	**Accessibility**	**Acceptability/ Patient-centeredness**	**Safety**	**Equity**
**Donabedian Framework**	**Structure**	- Mean percent score of ANC key services offered.	- ANC service readiness.<br>- Whether family planning counseling is routinely conducted during ANC visit.	- Number of days per month ANC services are provided.<br>- Availability of folic acid on day of interview.<br>- Available and functional ANC equipment and supplies score.<br>- Availability of medications/supplies necessary to provide evidence-based essential maternal health care.	- Availability of visual aids for client education related to pregnancy/ ANC	- Infection prevention score.<br>- ANC visit conducted by qualified provider.	-
	**Process**	- Physical exam score for services observed during first time ANC visits.	- Pre-ANC consultation services score (services observed).	-	- Assured privacy during ANC consult.	-	-
	**Outcome**	-	-	-	- Patient satisfaction	-	-

Abbreviations: ANC – antenatal care.

Table S3. Unweighted characteristics of surveyed individuals, clusters, and regions included in the intermittent preventive treatment in pregnancy analysis.

Variables	Total (n=15175) n (%)/ median (IQR)	Kenya (n=7861) n (%)/ median (IQR)	Namibia (n=1639) n (%)/ median (IQR)	Senegal (n=2682) n (%)/ median (IQR)	Tanzania (n=2993) n (%)/ median (IQR)
**Level 1 - Individual**					
IPTp uptake during pregnancy (yes)	3516 (23.17)	1409 (17.92)	69 (4.21)	1048 (39.08)	990 (33.08)
Mother's age in years	27 (22, 32)	26 (22, 31)	27 (22, 33)	27 (23, 33)	27 (23, 33)
Mother's education (none)	4422 (29.14)	1681 (21.38)	123 (7.50)	1865 (69.54)	753 (25.16)
Primary	6850 (45.14)	4054 (51.57)	386 (23.55)	555 (20.69)	1855 (61.98)
Secondary or higher	3903 (25.72)	2126 (27.04)	1130 (68.94)	262 (9.77)	385 (12.86)
Parity	3 (2, 5)	3 (2, 5)	2 (1, 4)	3 (2, 5)	3 (2, 5)
Number of ANC visits during last live birth	4 (3, 5)	4 (3, 5)	5 (4, 7)	3 (2, 4)	3 (3, 4)
Household wealth quintile (poorest)	4488 (29.57)	2765 (35.17)	362 (22.09)	761 (28.37)	600 (20.05)
Poorer	3328 (21.93)	1605 (20.42)	343 (20.93)	696 (25.95)	684 (22.85)
Middle	2857 (18.83)	1300 (16.54)	359 (21.90)	578 (21.55)	620 (20.72)
Richer	2489 (16.40)	1153 (14.67)	333 (20.32)	385 (14.35)	618 (20.65)
Richest	2013 (13.27)	1038 (13.20)	242 (14.77)	262 (9.77)	471 (15.74)
Household size	6 (4, 9)	5 (4, 7)	6 (4, 8)	13 (9, 19)	6 (5, 9)
**Level 2 - Survey cluster**					
Survey administered during malaria season (yes)	10336 (68.11)	5006 (63.68)	1101 (61.68)	1326 (49.44)	2993 (100.00)
Malaria endemicity	6.69 (2.84, 13.03)	9.02 (4.50, 18.91)	5.17 (0.00,7.64)	2.54 (2.05, 3.69)	7.13 (4.34, 13.68)
Residence location (urban)	4615 (30.41)	2539 (32.30)	729 (44.48)	784 (29.23)	563 (18.81)
**Level 3 - Region**					
Antenatal care quality	74.56 (70.03, 76.90)	76.32 (74.56, 79.18)	73.05 (69.59, 74.35)	73.56 (71.23, 76.64)	57.83 (53.70, 63.26)
Malaria in pregnancy quality	52.94 (41.94, 54.51)	54.47 (53.32, 54.51)	30.18 (16.95, 35.64)	44.51 (42.81, 47.05)	42.26 (38.97, 46.33)
Facility density per 1,000,000 population	14.62 (9.02, 20.96)	12.01 (6.41, 14.64)	131.14 (86.12, 184.40)	24.60 (20.53, 32.14)	9.30 (6.48, 13.91)
Prevalence of HIV in women 15-49 years	6.3 (2.4, 8.5)	6.3 (5.8, 9.2)	15 (12.2, 20.3)	1.2 (0.5, 1.8)	5.7 (2.3, 7.4)

Abbreviations: n–count; IQR– interquartile range; IPTp–intermittent preventive treatment in pregnancy.

Table S4. Unweighted characteristics of surveyed individuals, clusters, and regions included in the insecticide-treated net use in pregnancy analysis.

Variables	Total (n=2378) n (%)/ median (IQR)	Kenya (n=662) n (%)/ median (IQR)	Namibia (n=207) n (%)/ median (IQR)	Senegal (n=729) n (%)/ median (IQR)	Tanzania (n=780) n (%)/ median (IQR)
**Level 1 - Individual**					
ITN use the night prior in pregnancy (yes)	1408 (59.21)	470 (71.00)	23 (11.11)	400 (54.87)	515 (66.03)
Mother's age in years	26 (22, 31)	26 (22, 31)	26 (21, 33)	26 (22, 32)	26 (22, 31)
Mother's education (none)	827 (34.78)	103 (15.56)	22 (10.63)	507 (69.55)	195 (25.00)
Primary	1012 (42.56)	339 (51.21)	43 (20.77)	150 (20.58)	480 (61.54)
Secondary or higher	539 (22.67)	220 (33.23)	142 (68.60)	72 (9.88)	105 (13.46)
Parity	2 (1, 4)	2 (1, 3)	1 (0, 3)	2 (1, 4)	2 (1, 4)
Gestational age	5 (3, 7)	5 (3, 7)	6 (4, 7)	6 (4, 7)	5 (3, 7)
Household wealth quintile (poorest)	545 (22.92)	170 (25.68)	40 (19.32)	218 (29.90)	117 (15.00)
Poorer	578 (24.31)	145 (21.90)	51 (24.64)	185 (25.38)	197 (25.26)
Middle	507 (21.32)	122 (18.43)	44 (21.26)	156 (21.40)	185 (23.72)
Richer	460 (19.34)	123 (18.58)	55 (26.57)	108 (14.81)	174 (22.31)
Richest	288 (12.11)	102 (15.41)	17 (8.21)	62 (8.50)	107 (13.72)
Household size	6 (4, 11)	4 (3, 6)	5 (4, 8)	13 (9, 18)	6 (4, 9)
**Level 2 - Survey cluster**					
Survey administered during malaria season (yes)	1791 (75.32)	477 (72.05)	165 (79.71)	369 (50.62)	780 (100.00)
Malaria endemicity	6.16 (2.65, 12.55)	11.03 (5.90, 21.23)	6.55 (2.37, 7.86)	2.46 (1.96, 3.39)	8.12 (4.39, 14.95)
Residence location (urban)	656 (27.59)	243 (36.71)	83 (40.10)	209 (28.67)	121 (15.51)
**Level 3 - Region**					
Antenatal care quality	73.37 (63.66, 76.64)	76.32 (74.56, 79.18)	74.24 (70.03, 77.79)	75.09 (71.23, 76.64)	56.64 (53.70, 63.66)
Malaria in pregnancy quality	46.33 (41.38, 53.32)	54.47 (53.32, 54.51)	35.64 (30.18, 38.87)	44.51 (42.81, 47.05)	42.70 (38.97, 46.33)
Facility density per 1,000,000 population	15.54 (9.30, 28.05)	12.01 (6.41, 15.54)	96.38 (86.12, 184.40)	24.60 (20.53, 33.34)	9.16 (6.48, 13.91)
Prevalence of HIV in women 15-49 years	4.10 (0.90, 8.40)	6.30 (5.80, 9.20)	20.30 (14.20, 22.10)	1.10 (0.50, 1.80)	5.70 (2.30, 8.40)

Abbreviations: n– count; IQR–interquartile range; ITN–insecticide-treated bed net.

Table S5. Unweighted characteristics of surveyed individuals, clusters, and regions included in the insecticide-treated net use in children under five analyses.

Variables	Total (n=27217) n (%)/ median (IQR)	Kenya (n=13870) n (%)/ median (IQR)	Namibia (n=1570) n (%)/ median (IQR)	Senegal (n=5602) n (%)/ median (IQR)	Tanzania (n=6175) n (%)/ median (IQR)
**Level 1 - Individual**					
ITN use the night prior in children under-5 (yes)	17795 (65.38)	10073 (72.62)	250 (15.92)	3172 (56.62)	4300 (69.64)
Child's age in months	28 (13, 43)	28 (13, 43)	25 (11, 40)	27 (13, 43)	27 (13, 43)
Child's gender (female)	13554 (49.80)	6872 (49.55)	780 (49.68)	2795 (49.89)	3107 (50.32)
Mother's age in years	28 (24, 34)	28 (24, 33)	29 (24, 35)	28 (24, 34)	29 (24, 35)
Mother's education (none)	7743 (28.45)	2055 (14.82)	104 (6.62)	4012 (71.62)	1572 (25.46)
Primary	13127 (48.23)	7750 (55.88)	346 (22.04)	1153 (20.58)	3878 (62.80)
Secondary or higher	6347 (23.32)	4065 (29.31)	1120 (71.34)	437 (7.80)	725 (11.74)
Parity	3 (2, 5)	3 (2, 5)	3 (2, 4)	4 (2, 6)	4 (2, 6)
Number of ANC visits during last live birth	4 (3, 5)	4 (3, 5)	6 (4, 8)	3 (2, 4)	3 (3, 4)
% Missing	*8072 (29.66)*	*3754 (27.07)*	*540 (34.39)*	*1873 (33.43)*	*1905 (30.85)*
Household wealth quintile (poorest)	6942 (25.51)	3797 (27.38)	396 (25.22)	1576 (28.13)	1173 (19.00)
Poorer	6418 (23.58)	3141 (22.65)	371 (23.63)	1487 (26.54)	1419 (22.98)
Middle	5444 (20.00)	2514 (18.13)	367 (23.38)	1232 (21.99)	1331 (21.55)
Richer	4692 (17.24)	2268 (16.35)	271 (17.26)	843 (15.05)	1310 (21.21)
Richest	3721 (13.67)	2150 (15.50)	165 (10.51)	464 (8.28)	942 (15.26)
Household size	6 (5, 9)	5 (4, 7)	6 (4, 8)	13 (9, 19)	6 (5, 9)
**Level 2 - Survey cluster**					
Survey administered during malaria season (yes)	19845 (72.91)	9647 (69.55)	1119 (71.27)	2904 (51.84)	6175 (100.00)
Malaria endemicity	7.30 (3.07, 15.45)	11.09 (5.99, 21.38)	6.24 (1.83, 7.79)	2.46 (1.99, 3.39)	7.40 (4.35, 14.29)
Residence location (urban)	8131 (29.87)	4865 (35.08)	542 (34.52)	1607 (28.69)	1117 (18.09)
**Level 3 - Region**					
Antenatal care quality	74.56 (69.82, 76.90)	76.32 (74.56, 79.18)	73.86 (69.59, 76.10)	73.56 (71.23, 76.64)	57.83 (53.70, 63.66)
Malaria in pregnancy quality	52.94 (42.81, 54.51)	54.47 (53.32, 54.51)	33.45 (27.64, 37.13)	46.38 (42.81, 50.00)	42.26 (38.97, 46.33)
Facility density per 1,000,000 population	13.91 (9.13, 20.53)	12.01 (6.41, 15.54)	130.61 (92.90, 184.40)	24.60 (20.53, 33.34)	9.30 (6.48, 13.91)
Prevalence of HIV in women 15-49 years	5.8 (1.8, 8.4)	6.3 (5.8, 9.2)	19.8 (14.2, 22.1)	1.2 (0.5, 1.8)	5.7 (2.3, 8.4)

Abbreviations: n–count; IQR– interquartile range; ITN–insecticide-treated bed net.

Table S6. Unadjusted pooled and by country risk estimates for factors associated with receipt of two or more doses of intermittent preventive treatment in pregnancy during last live birth in preceding 24 months.

	**Kenya (n=7913)**		**Namibia (n=2051)**		**Senegal (n=2710)**		**Tanzania (n=3156)**		**Pooled (n=15175)**	
**Measures of Association**	**RR**	**95% CI**	**RR**	**95% CI**	**RR**	**95% CI**	**RR**	**95% CI**	**RR**	**95% CI**
Individual Level										
Mother's age	1.002	(0.992, 1.013)	0.989	(0.953, 1.027)	0.999	(0.991, 1.008)	1.007	(0.997, 1.016)	1.005	(0.999, 1.011)
Mother's education (none)	Ref	Ref	Ref	Ref	Ref	Ref	Ref	Ref	Ref	Ref
Primary	1.000	(0.841, 1.190)	2.347	(0.527, 10.446)	**1.299**	**(1.140, 1.479)**	**1.219**	**(1.023, 1.452)**	**0.822**	**(0.753, 0.896)**
Secondary or higher	0.834	(0.689, 1.010)	2.510	(0.604, 10.431)	1.158	(0.948, 1.414)	**1.799**	**(1.413, 2.290)**	**0.522**	**(0.462, 0.590)**
Parity	**1.033**	**(1.003, 1.063)**	0.895	(0.749, 1.071)	**0.964**	**(0.940, 0.988**	0.979	(0.951, 1.007)	1.008	(0.991, 1.026)
# of ANC visits	**1.115**	**(1.085, 1.146)**	1.037	(0.976, 1.102)	**1.189**	**(1.141, 1.239)**	**1.167**	**(1.122, 1.214)**	0.997	(0.983, 1.012)
Household size	**1.052**	**(1.028, 1.077)**	1.043	(0.978, 1.113)	1.004	(0.997, 1.010)	**0.972**	**(0.953, 0.990)**	**1.031**	**(1.026, 1.035)**
Wealth quintile (Poorest)	Ref	Ref	Ref	Ref	Ref	Ref	Ref	Ref	Ref	Ref
Poorer	**0.835**	**(0.702, 0.992)**	0.591	(0.260, 1.344)	**1.243**	**(1.033, 1.496)**	0.958	(0.775, 1.184)	1.044	(0.927, 1.176)
Middle	1.021	(0.862, 1.210)	1.181	(0.621, 2.248)	**1.594**	**(1.343, 1.893)**	1.078	(0.875, 1.329)	**1.283**	**(1.146, 1.438)**
Richer	**0.691**	**(0.573, 0.833)**	0.919	(0.448, 1.884)	**1.635**	**(1.350, 1.980)**	1.138	(0.915, 1.417)	**1.185**	**(1.043, 1.346)**
Richest	**0.659**	**(0.537, 0.810)**	0.456	(0.185, 1.121)	**1.867**	**(1.528, 2.281)**	**1.282**	**(1.026, 1.602)**	**1.297**	**(1.131, 1.489)**
Location x MIP quality[1,2]	**1.221**	**(1.175, 1.269)**	1.007	(0.948, 1.069)	0.992	(0.971, 1.013)	0.992	(0.976, 1.009)	**1.016**	**(1.009, 1.022)**
Location x ANC quality[1,2]	**0.870**	**(0.842, 0.899)**	1.123	(0.947, 1.333)	1.013	(0.979, 1.048)	1.013	(0.981, 1.045)	**0.989**	**(0.980, 0.999)**
ANC x MiP quality[1,2]	**1.030**	**(1.026, 1.034)**	0.999	(0.992, 1.008)	0.999	(0.995, 1.003)	**0.998**	**(0.997, 0.999)**	**0.999**	**(0.998, 0.999)**
Cluster Level										
Residence location (urban)	**0.776**	**(0.677, 0.890)**	1.005	(0.614, 1.647)	**1.418**	**(1.261, 1.594)**	**1.199**	**(1.015, 1.417)**	1.085	(0.991, 1.188)
Malaria endemicity	**1.047**	**(1.042, 1.052)**	**1.110**	**(1.032, 1.195)**	**0.965**	**(0.950, 0.981)**	1.003	(0.994, 1.011)	1.003	(0.999, 1.008)
Malaria season (Yes)	2.833	(2.364, 3.395)	2.397	(1.233, 4.659)	1.075	(0.958, 1.205)	--	--	**1.138**	**(1.034, 1.253)**
Region Level										
ANC quality[3]	**1.050**	**(1.034, 1.066)**	1.053	(0.978, 1.132)	**0.978**	**(0.961, 0.994)**	1.007	(0.995, 1.020)	**0.994**	**(0.990, 0.998)**
MiP quality[3]	**1.034**	**(1.022, 1.045)**	**1.042**	**(1.010, 1.074)**	**0.988**	**(0.978, 0.999)**	**1.015**	**(1.006, 1.023)**	**1.021**	**(1.018, 1.024)**
Facility density[4]	1.211	(1.184, 1.239)	1.000	(0.997, 1.004)	0.999	(0.997, 1.002)	**1.014**	**(1.011, 1.017)**	**0.994**	**(0.993, 0.995)**
HIV prevalence[5]	**1.032**	**(1.019, 1.045)**	**1.030**	**(1.003, 1.058)**	**0.869**	**(0.808, 0.933)**	**0.960**	**(0.939, 0.981)**	**0.914**	**(0.906, 0.923)**
Country Level										
Country (Kenya)	**--**	**--**	**--**	**--**	**--**	**--**	**--**	**--**	Ref	Ref
Namibia	**--**	**--**	**--**	**--**	**--**	**--**	**--**	**--**	**0.296**	**(0.230, 0.382)**
Senegal	**--**	**--**	**--**	**--**	**--**	**--**	**--**	**--**	**2.464**	**(2.264, 2.681)**
Tanzania	**--**	**--**	**--**	**--**	**--**	**--**	**--**	**--**	**1.607**	**(1.465, 1.762)**

Abbreviations: n–count; RR–risk ratio; CI–confidence interval; Ref–reference level; MiP–malaria in pregnancy; ANC–antenatal care; HIV–human immunodeficiency virus.

[1] Calculated using mean-centered quality score(s). [2] Includes individual variables for each interaction term. [3] Mean-centered for each country. [4] per 1,000,000 population. [5] in reproductive-age women 15-49 years.

Table S7. Unadjusted pooled and by-country risk estimates for factors associated with use of insecticide-treated bed net the night prior in current pregnancy.

	Kenya (n=662)		Namibia (n=207)		Senegal (n=729)		Tanzania (n=780)		Pooled (n=2378)	
	**RR**	**95% CI**	**RR**	**95% CI**	**RR**	**95% CI**	**RR**	**95% CI**	**RR**	**95% CI**
**Measures of Association**										
Individual Level										
Mother's age	**1.021**	**(1.011, 1.032)**	1.024	(0.971, 1.079)	1.000	(0.986, 1.014)	1.007	(0.997, 1.016)	1.003	(0.996, 1.011)
Mother's education (none)	Ref	Ref	Ref	Ref	Ref	Ref	Ref	Ref	Ref	Ref
Primary	0.983	(0.831, 1.162)	0.776	(0.158, 3.809)	1.212	(0.981, 1.497)	1.037	(0.899, 1.197)	**1.230**	**(1.108, 1.366)**
Secondary or higher	0.900	(0.744, 1.090)	0.889	(0.218, 3.615)	1.266	(0.947, 1.692)	0.747	(0.533, 1.047)	**0.710**	**(0.601, 0.838)**
Parity	1.028	(0.990, 1.067)	1.130	(0.946, 1.349)	1.015	(0.979, 1.052)	**1.034**	**(1.011, 1.057)**	**1.036**	**(1.016, 1.056)**
Gestational age	1.018	(0.985, 1.052)	0.877	(0.722, 1.065)	0.981	(0.942, 1.021)	1.003	(0.976, 1.030)	0.993	(0.971, 1.015)
Household size	0.978	(0.945, 1.013)	0.946	(0.802, 1.115)	**0.987**	**(0.975, 0.998)**	0.987	(0.972, 1.002)	**0.986**	**(0.978, 0.994)**
Wealth quintile (Poorest)	Ref	Ref	Ref	Ref	Ref	Ref	Ref	Ref	Ref	Ref
Poorer	1.179	(0.983, 1.415)	1.152	(0.428, 3.102)	1.066	(0.826, 1.375)	1.040	(0.885, 1.221)	1.127	(0.981, 1.295)
Middle	1.112	(0.901, 1.373)	0.408	(0.094, 1.774)	**1.469**	**(1.173, 1.840)**	0.941	(0.785, 1.129)	**1.171**	**(1.018, 1.347)**
Richer	1.005	(0.802, 1.260)	0.475	(0.139, 1.621)	1.120	(0.823, 1.524)	0.962	(0.802, 1.153)	0.976	(0.829, 1.149)
Richest	0.967	(0.748, 1.251)	**0.112**	**(0.013, 0.965)**	1.109	(0.753, 1.633)	0.826	(0.649, 1.051)	0.997	(0.822, 1.211)
Location x MiP quality[1,2]	**1.047**	**(1.001, 1.094)**	**1.151**	**(1.050, 1.261)**	**1.049**	**(1.016, 1.083)**	0.988	(0.967, 1.009)	**1.027**	**(1.014, 1.039)**
Location x ANC quality[1,2]	**0.935**	**(0.897, 0.976)**	1.118	(0.695, 1.796)	0.944	(0.884, 1.009)	1.014	(0.976, 1.053)	1.005	(0.991, 1.020)
ANC quality x MiP quality[1,2]	**1.009**	**(1.003, 1.015)**	1.012	(0.991, 1.034)	1.006	(0.999, 1.013)	0.999	(0.999, 1.001)	1.007	(0.993, 1.021)
Cluster Level										
Residence location (urban)	0.987	(0.844, 1.155)	**0.262**	**(0.081, 0.846)**	0.952	(0.763, 1.188)	0.827	(0.680, 1.006)	**0.785**	**(0.689, 0.894)**
Malaria endemicity	**1.017**	**(1.010, 1.024)**	0.995	(0.901, 1.099)	0.984	(0.959, 1.010)	**1.009**	**(1.003, 1.015)**	**1.021**	**(1.017, 1.025)**
Malaria season (yes)	1.149	(0.973, 1.356)	0.600	(0.256, 1.407)	1.209	(1.004, 1.455)	--	--	**1.324**	**(1.159, 1.513)**
Region Level										
ANC quality[3]	0.990	(0.967, 1.013)	**0.815**	**(0.708, 0.937)**	**1.033**	**(1.002, 1.064)**	1.008	(0.997, 1.018)	**0.940**	**(0.980, 0.989)**
MiP quality[3]	1.024	(0.999, 1.048)	**1.040**	**(1.002, 1.080)**	**1.018**	**(1.002, 1.034)**	1.005	(0.997, 1.012)	**1.025**	**(1.020, 1.030)**
Facility density[4]	**1.028**	**(1.010, 1.046)**	**1.013**	**(1.007, 1.019)**	0.999	(0.996, 1.004)	0.995	(0.989, 1.000)	**0.990**	**(0.987, 0.993)**
HIV prevalence[5]	1.008	(0.994, 1.023)	**1.177**	**(1.114, 1.244)**	1.001	(0.894, 1.120)	0.980	(0.960, 1.001)	**0.972**	**(0.964, 0.981)**
Country Level										
Country (Kenya)	--	--	--	--	--	--	--	--	Ref	Ref
Namibia	--	--	--	--	--	--	--	--	**0.141**	**(0.093, 0.215)**
Senegal	--	--	--	--	--	--	--	--	**0.729**	**(0.649, 0.819)**
Tanzania	--	--	--	--	--	--	--	--	0.986	(0.898, 1.083)

Abbreviations: n–count; RR–risk ratio; CI–confidence interval; Ref–reference level; MiP–malaria in pregnancy; ANC–antenatal care; HIV–human immunodeficiency virus.

[1] Calculated using mean-centered quality score(s). [2] Includes individual variables for each interaction term. [3] Mean-centered for each country. [4] per 1,000,000 population. [5] in reproductive-age women 15-49 years.

Table S8. Unadjusted pooled and by-country risk estimates for factors associated with use of insecticide-treated bed net the night prior in children under five.

	Kenya (n=13870)		Namibia (n=1570)		Senegal (n=5602)		Tanzania (n=6175)		Pooled (n=27217)	
**Measures of Association**	**RR**	**95% CI**	**RR**	**95% CI**	**RR**	**95% CI**	**RR**	**95% CI**	**RR**	**95% CI**
Individual Level										
Child's age in months	**0.997**	**(0.996, 0.998)**	**0.990**	**(0.983, 0.997)**	0.999	(0.997, 1.000)	0.999	(0.999, 1.001)	**0.999**	**(0.998, 0.999)**
Child's sex (Female)	**0.955**	**(0.930, 0.982)**	0.901	(0.704, 1.152)	0.984	(0.926, 1.044)	1.008	(0.971, 1.046)	0.979	(0.953, 1.006)
Mother's age in years	0.999	(0.997, 1.001)	0.997	(0.980, 1.015)	1.002	(0.997, 1.006)	0.999	(0.996, 1.002)	**0.998**	**(0.996, 0.999)**
Mother's education (none)	Ref	Ref	Ref	Ref	Ref	Ref	Ref	Ref	Ref	Ref
Primary	**1.096**	**(1.043, 1.152)**	1.918	(0.816, 4.510)	1.009	(0.933, 1.091)	1.032	(0.986, 1.080)	**1.145**	**(1.110, 1.181)**
Secondary or higher	**1.212**	**(1.151, 1.275)**	2.201	(0.965, 5.021)	**1.151**	**(1.033, 1.284)**	0.971	(0.891, 1.059)	**0.897**	**(0.860, 0.936)**
Parity	**0.981**	**(0.975, 0.987)**	0.967	(0.910, 1.027)	0.997	(0.985, 1.009)	0.996	(0.989, 1.004)	1.002	(0.996, 1.007)
# of mother's ANC visits for last live birth	1.009	(0.999, 1.018)	**0.951**	**(0.915, 0.989)**	**1.053**	**(1.028, 1.078)**	1.003	(0.988, 1.018)	**0.960**	**(0.951, 0.968)**
Household size	**0.970**	**(0.964, 0.976)**	**0.943**	**(0.899, 0.988)**	**0.985**	**(0.981, 0.989)**	**0.986**	**(0.980, 0.991)**	**0.983**	**(0.980, 0.985)**
Wealth quintile (poorest)	Ref	Ref	Ref	Ref	Ref	Ref	Ref	Ref	Ref	Ref
Poorer	**1.128**	**(1.085, 1.173)**	0.899	(0.654, 1.237)	**1.278**	**(1.169, 1.397)**	0.963	(0.913, 1.016)	**1.122**	**(1.078, 1.168)**
Middle	**1.148**	**(1.102, 1.196)**	**0.556**	**(0.388, 0.797)**	**1.512**	**(1.391, 1.644)**	0.949	(0.897, 1.003)	**1.154**	**(1.108, 1.202)**
Richer	**1.166**	**(1.115, 1.219)**	0.735	(0.507, 1.064)	**1.389**	**(1.258, 1.533)**	0.994	(0.940, 1.051)	**1.185**	**(1.135, 1.237)**
Richest	**1.148**	**(1.096, 1.202)**	0.670	(0.414, 1.085)	**1.254**	**(1.096, 1.436)**	**0.921**	**(0.861, 0.984)**	**1.164**	**(1.109, 1.222)**
Location x MiP quality[1,2]	**1.023**	**(1.014, 1.032)**	**1.048**	**(1.004, 1.094)**	1.009	(0.999, 1.019)	1.003	(0.998, 1.008)	**1.009**	**(1.006, 1.013)**
Location x ANC quality[1,2]	**0.970**	**(0.962, 0.978)**	1.030	(0.958, 1.108)	**0.918**	**(0.901, 0.936**	0.999	(0.989, 1.008)	**1.007**	**(1.004, 1.010)**
ANC quality x MiP quality[1,2]	**1.004**	**(1.003, 1.005)**	1.001	(0.995, 1.006)	**1.007**	**(1.005, 1.009)**	**1.001**	**(1.000, 1.001)**	**1.002**	**(1.002, 1.002)**
Child's sex x MiP quality[1,2]	**1.012**	**(1.003, 1.021)**	0.996	(0.955, 1.038)	0.998	(0.988, 1.008)	0.999	(0.995, 1.004)	0.999	(0.997, 1.003)
Child's sex x ANC quality[1,2]	0.992	(0.983, 1.001)	0.991	(0.929, 1.058)	0.995	(0.976, 1.014)	0.999	(0.993, 1.006)	**0.997**	**(0.995, 0.999)**
Child's age x MiP quality[1,2]	0.999	(0.999, 1.000)	1.000	(0.999, 1.002)	0.999	(0.999, 1.000)	0.999	(0.999, 1.000)	**0.999**	**(0.999, 0.999)**
Child's age x ANC quality[1,2]	1.000	(0.999, 1.000)	0.998	(0.997, 1.000)	0.999	(0.999, 1.000)	0.999	(0.999, 1.000)	**0.999**	**(0.999, 0.999)**
Cluster Level										
Residence location (urban)	**1.047**	**(1.015, 1.079)**	1.068	(0.822, 1.387)	**1.137**	**(1.062, 1.217)**	0.964	(0.915, 1.015)	1.011	(0.980, 1.044)
Malaria endemicity	**1.006**	**(1.005, 1.008)**	1.003	(0.969, 1.039)	**0.967**	**(0.958, 0.977)**	**1.007**	**(1.005, 1.009)**	**1.016**	**(1.015, 1.017)**
Malaria season (yes)	**1.127**	**(1.093, 1.163)**	**0.767**	**(0.593, 0.993)**	**1.149**	**(1.082, 1.221)**	--	--	**1.224**	**(1.183, 1.267)**
Region Level										
ANC quality[3]	1.002	(0.997, 1.006)	**0.923**	**(0.894, 0.954)**	**1.027**	**(1.017, 1.037)**	0.999	(0.997, 1.003)	**0.993**	**(0.992, 0.994)**
MiP quality[3]	**1.006**	**(1.002, 1.011)**	**1.037**	**(1.015, 1.059)**	**1.015**	**(1.010, 1.020)**	**1.003**	**(1.000, 1.005)**	**1.022**	**(1.021, 1.024)**
Facility density[4]	**1.016**	**(1.013, 1.020)**	**1.007**	**(1.004, 1.009)**	**0.998**	**(0.997, 0.999)**	**0.996**	**(0.994, 0.998)**	**0.992**	**(0.991, 0.993)**
HIV prevalence[5]	**1.007**	**(1.004, 1.009)**	**1.079**	**(1.061, 1.097)**	**0.956**	**(0.920, 0.993)**	0.999	(0.993, 1.004)	**0.981**	**(0.979, 0.984)**
Country Level										
Country (Kenya)	**--**	**--**	**--**	**--**	**--**	**--**	**--**	**--**	Ref	Ref
Namibia	**--**	**--**	**--**	**--**	**--**	**--**	**--**	**--**	**0.223**	**(0.197, 0.252)**

Senegal	--	--	--	--	--	--	--	--	**0.752**	**(0.728, 0.777)**
Tanzania	--	--	--	--	--	--	--	--	0.991	(0.968, 1.014)

Abbreviations: n–count; RR–risk ratio; CI–confidence interval; Ref–reference level; MiP–malaria in pregnancy; ANC– antenatal care; HIV–human immunodeficiency virus.

[1] Calculated using mean-centered quality score(s).

[2] Includes individual variables for each interaction term.

[3] Mean-centered for each country.

[4] per 1,000,000 population.

[5] in reproductive-age women 15-49 years.

Supplemental References

1. World Health Organization. *Quality of Care: A process for making strategic choices in health systems*; World Health Organization: Geneva, Switzerland, 2006 2006.
2. Donabedian, A. The quality of care. How can it be assessed? *JAMA* **1988**, *260*, 1743-1748.
3. Lee, E.; Madhavan, S.; Bauhoff, S. Levels and variations in the quality of facility-based antenatal care in Kenya: evidence from the 2010 service provision assessment. *Health Policy and Planning* **2016**, *31*, 777-784, doi:10.1093/heapol/czv132.
4. Maheu-Giroux, M.; Castro, M.C. Factors affecting providers' delivery of intermittent preventive treatment for malaria in pregnancy: a five-country analysis of national service provision assessment surveys. *Malaria Journal* **2014**, *13*, 440, doi:10.1186/1475-2875-13-440.
5. Hill, J.; Kazembe, P. Reaching the Abuja target for intermittent preventive treatment of malaria in pregnancy in African women: a review of progress and operational challenges. *Trop Med Int Health* **2006**, *11*, 409-418, doi:10.1111/j.1365-3156.2006.01585.x.
6. Gross, K.; Alba, S.; Schellenberg, J.; Kessy, F.; Mayumana, I.; Obrist, B. The combined effect of determinants on coverage of intermittent preventive treatment of malaria during pregnancy in the Kilombero Valley, Tanzania. *Malar J* **2011**, *10*, 140, doi:10.1186/1475-2875-10-140.
7. Anders, K.; Marchant, T.; Chambo, P.; Mapunda, P.; Reyburn, H. Timing of intermittent preventive treatment for malaria during pregnancy and the implications of current policy on early uptake in north-east Tanzania. *Malar J* **2008**, *7*, 79, doi:10.1186/1475-2875-7-79.
8. Marchant, T.; Nathan, R.; Jones, C.; Mponda, H.; Bruce, J.; Sedekia, Y.; Schellenberg, J.; Mshinda, H.; Hanson, K. Individual, facility and policy level influences on national coverage estimates for intermittent preventive treatment of malaria in pregnancy in Tanzania. *Malar J* **2008**, *7*, 260, doi:10.1186/1475-2875-7-260.
9. Zou, G. A Modified Poisson Regression Approach to Prospective Studies with Binary Data. *American Journal of Epidemiology* **2004**, *159*, 702-706, doi:10.1093/aje/kwh090.
10. StataCorpLP. Stata Multilevel Mixed-Effects Reference Manual Release 14. **2015**.
11. Analyzing Correlated (Clustered) Data. Available online: http://www.ats.ucla.edu/stat/stata/library/cpsu.htm (accessed on
12. Royston, P.; Sauerbrei, W. Handling interactions in Stata Handling interactions in Stata, especially with continuous predictors. In Proceedings of the German Stata Users' meeting, Berlin, Germany, 1 June 2012, 2012.
13. Moineddin, R.; Matheson, F.I.; Glazier, R.H. A simulation study of sample size for multilevel logistic regression models. *BMC Med Res Methodol* **2007**, *7*, 34, doi:10.1186/1471-2288-7-34.
14. Ferguson, C. An Effect Size Primer: A Guide for Clinicians and Researchers. *Professional Psychology: Research and Practice* **2009**, *40*, 532–538.
15. Peduzzi, P.; Concato, J.; Kemper, E.; Holford, T.R.; Feinstein, A.R. A simulation study of the number of events per variable in logistic regression analysis. *J Clin Epidemiol* **1996**, *49*, 1373-1379.
16. Green, S.B. How Many Subjects Does it Take to do a Regression Analysis? *Multivariate Behavioral Research* **1991**, *26*, 499-510.
